# Present‐day sympatry belies the evolutionary origin of a high‐order polyploid

**DOI:** 10.1111/nph.14711

**Published:** 2017-08-03

**Authors:** Na Wei, Jacob A. Tennessen, Aaron Liston, Tia‐Lynn Ashman

**Affiliations:** ^1^ Department of Biological Sciences University of Pittsburgh Pittsburgh PA 15260 USA; ^2^ Department of Integrative Biology Oregon State University Corvallis OR 97331 USA; ^3^ Department of Botany and Plant Pathology Oregon State University Corvallis OR 97331 USA

**Keywords:** decaploid *Fragaria*, linkage map, phylogenetics, polyploid origin, sex expression, subgenome, targeted sequence capture

## Abstract

- Disentangling the evolutionary histories of polyploids, especially those with high ploidies, can reveal fundamental processes in speciation. Despite occurring frequently during evolution, the origins of many extant polyploid plant species remain largely unknown.
- By integrating linkage mapping, polyploid phylogeny and sex‐determining region (SDR) in a unified framework, we statistically evaluated evolutionary hypotheses concerning the origin of a recently recognized decaploid strawberry (*Fragaria cascadensis*).
- The maximum‐likelihood phylogenies and topology tests across homeologous groups consistently rejected the seemingly parsimonious hypothesis of ‘contemporary sympatric speciation’ via hybridization between octoploid and diploid congeners. Instead, most chromosomes supported ‘ancient hybrid speciation’ between a maternal octoploid progenitor ancestral to extant octoploid strawberries and a paternal, extinct *Fragaria iinumae*‐like diploid progenitor, probably in Beringia during the Pleistocene. The absence of a shared SDR between the decaploid and other *Fragaria* is also consistent with an older origin rather than a recent hybrid origin *in situ*.
- Our study reveals a long evolutionary history of the decaploid despite its recent discovery, and highlights the pitfalls of inferring polyploid origins from niche/range alone or combined with morphology. It can serve as an exemplary starting step towards building much‐needed model systems of established polyploids that have been, and remain to be, recognized.

## Introduction

Polyploidy, whole‐genome duplication often with hybridization, creates organisms that possess more than two sets of chromosomes of the same or disparate origins (Otto & Whitton, 2000; Comai, 2005; Soltis & Soltis, 2009). Paleopolyploidy events that occurred at the crown and deep branches of angiosperms have revealed that all flowering plants have a polyploid ancestry (Jaillon *et al*., 2007; Tang *et al*., 2010; Jiao *et al*., 2011; Vanneste *et al*., 2014). In addition to paleopolyploids, the widespread incidence of extant polyploid species has also been documented, accounting for one‐quarter to one‐third of vascular plants (Wood *et al*., 2009; Barker *et al*., 2016). However, with the exception of a few polyploid‐rich genera and economic crops, we know little about the origins of these contemporary polyploids (Soltis *et al*., 2014, 2016). In particular, compared with low‐order (2*n* = 4*x*) and recently formed neopolyploids (< 150 yr; reviewed in Soltis *et al*., 2016), this knowledge especially from a genome‐wide perspective remains scant for high‐order (2*n* ≥ 6*x*) and older extant polyploids (0.2–5 Myr), which probably have complicated evolutionary histories.

The genomes of high‐order polyploids can be complex and diverse for many reasons. Not only may they originate from the same progenitor species (autopolyploidy) or different species (allopolyploidy), but a given polyploid species can be formed independently and repeatedly in space (e.g. *Tragopogon*; Soltis *et al*., 2004; Symonds *et al*., 2010), acting as a diversity‐enhancing mechanism in the evolution of polyploid genomes. Furthermore, the degree of relatedness of progenitors in allopolyploid formation can affect genomic inheritance and subgenome retention. For instance, when progenitor species are closely related, allopolyploids can functionally behave as autopolyploids, displaying polysomic inheritance (Sybenga, 1996; Ramsey & Schemske, 1998). The resulting distorted segregation can lead to unbalanced retention of subgenomes from different progenitor species (Doyle & Egan, 2010).

High‐order polyploids that acquire origins over geologic time from multiple progenitor species present a challenge to deciphering their evolutionary histories. This is not only because polyploid formation may involve hybridization within and between populations, progenitor species and ploidies over variable timeframes (e.g. Marcussen *et al*., 2014), but also because one or more progenitor species may go extinct, potentially confounding the inference of polyploid ancestries (Doyle & Egan, 2010). It is not uncommon for extant polyploid plant species to have originated from extinct progenitors (e.g. Roelofs *et al*., 1997; Jakob & Blattner, 2010). Dynamic genome restructuring since the onset of polyploidy (Gaeta *et al*., 2007; Leitch & Leitch, 2008; Xiong *et al*., 2011; Chester *et al*., 2012) adds another dimension of complexity to inferring origins of high‐order and old polyploids. For instance, homeologous exchange and chromosome substitution have been frequently detected in synthetic and natural neopolyploids (e.g. Xiong *et al*., 2011; Chester *et al*., 2012). However, the extent to which these immediate genomic changes persist remains largely unknown (Lim *et al*., 2007; Mavrodiev *et al*., 2015). As many aspects of genome rearrangements can lead to discordance between genes in phylogenetic signatures, the extent and duration of such changes affect the inference of polyploid origins.

A final complicating factor in inferring the origins of polyploids is the potential spatial mismatch between the contemporary distributions of a polyploid and its progenitor species. Spatial and/or niche segregation with progenitors are predicted to underlie the successful establishment and persistence of new polyploid lineages (Fowler & Levin, 1984, 2016; Levin, 2003; Oswald & Nuismer, 2011). First, although polyploidy can enforce instantaneous speciation in sympatry as a consequence of postzygotic barriers imposed between ploidal levels (Rieseberg & Willis, 2007), such reproductive isolation is not as complete as previously recognized, especially between high‐order polyploids (e.g. Sutherland & Galloway, 2017). Gene flow from high‐frequency parental species to nascent polyploids can slow their divergence. Second, demographic stochasticity or competition with abundant, locally adapted parental species can impede the persistence of new polyploids. These factors are most pronounced when polyploids exhibit niche similarity to progenitor species as a consequence of parental dominance or additivity. Polyploids that benefit from heterosis or transgressive phenotypes (Chen, 2010; Yoo *et al*., 2014) can, however, readily diverge in ecological niches and persist in novel environments relative to progenitors (te Beest *et al*., 2012). Therefore, present‐day sympatry of polyploids with diploids may not reflect a descendant–progenitor relationship; rather, it could represent niche complementarity with nonprogenitor diploid species, or recent contact and ongoing speciation. The incomplete insights gained from contemporary range information alone emphasize the need for genomic and phylogenetic contexts for assessing a polyploid origin.

Genome‐wide phylogenetic inference of polyploid origins entails distinguishing homologous and homeologous chromosomes or genes. This has traditionally been accomplished by the use of cytogenetic tools of genomic *in situ* hybridization (GISH) and fluorescence *in situ* hybridization (FISH) (e.g. Lim *et al*., 2000) or sequence similarity‐based orthologous variant discrimination (e.g. Marcussen *et al*., 2014). However, the ability to distinguish between homeologs can be hindered by various factors, such as similarity between subgenomes (or diploid progenitors) and insufficient representation of homeologous sequences, particularly in the cases of high‐order polyploids. These difficulties can, however, be circumvented by a linkage mapping‐assisted approach for homeolog identification and subgenome inference (e.g. Polimaps; Tennessen *et al*., 2014), in which the origin of individual chromosomes can be inferred by placing them in a phylogenetic tree with candidate progenitor species.

Wild strawberries (*Fragaria*) offer a model system for studying speciation and lineage persistence of high‐order polyploids. Within the past 3–8 Myr (Liston *et al*., 2014; Qiao *et al*., 2016), *Fragaria* diversified to encompass extensive ploidy variation (from diploids (2*x*) to decaploids (10*x*)) and broad geographic ranges (Liston *et al*., 2014). Approximately half of the extant 22 *Fragaria* species are polyploids, and these can occur both in sympatry and in allopatry with diploids (Staudt, 1999; Johnson *et al*., 2014; Liston *et al*., 2014). Reproductive isolation is often not yet complete between species, particularly for high‐order polyploids (Liston *et al*., 2014). For instance, two sister octoploid (8*x*) species, *Fragaria chiloensis* and *Fragaria virginiana*, hybridize naturally when in sympatry, producing the wild relative of the cultivated octoploid strawberry *Fragaria* ×*ananassa* ssp. *cuneifolia* (Salamone *et al*., 2013). Linkage mapping‐assisted characterization of the octoploid genomes (*F*. *chiloensis* and *F*. *virginiana* ssp. *virginiana*; Tennessen *et al*., 2014; cultivated strawberry *F*. ×*ananassa*; Sargent *et al*., 2016) have revealed the presence of one subgenome (Av) from diploid *Fragaria vesca*, one subgenome (Bi) from diploid *Fragaria iinumae*, and two subgenomes (B1 and B2) from unknown, extinct *Fragaria* *iinumae*‐like diploids. Physical chromosomal evidence (FISH and GISH; Liu *et al*., 2016) has confirmed this composition.

**Figure 1:**
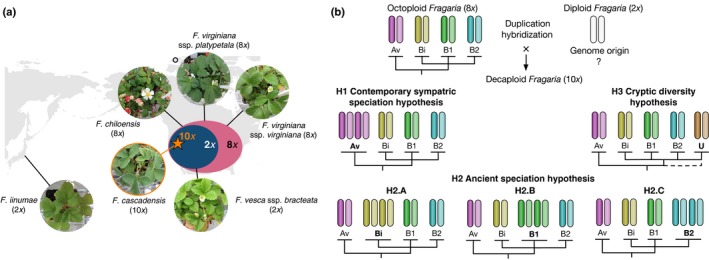
Hypotheses concerning the evolutionary origin of the recently discovered decaploid (10*x*) strawberry *Fragaria cascadensis*. (a) *Fragaria* *cascadensis* (denoted as a star) is endemic to the High Cascades, Oregon, USA, in sympatry with the octoploid (8*x*) *Fragaria virginiana* ssp. *platypetala* and the diploid (2*x*) *Fragaria vesca* ssp. *bracteata*, giving rise to the hypothesis of 8*x*–2*x* contemporary sympatric speciation for the formation of the decaploid. Other potential progenitors include the 8*x F*. *virginiana* ssp. *virginiana* (distributed broadly in eastern North America) and *Fragaria chiloensis* (distributed along the Pacific coast), as well as the allopatric 2*x Fragaria iinumae* (distributed in Japan and Sakhalin Island, Russia). The two ellipses roughly depict the present distributions of the 8*x* species and the 2*x F*. *vesca* ssp. *bracteata* in the USA. The black circle denotes the locality where the only fossil record of *Fragaria* was documented (Liston *et al*., 2014). (b) Chromosomal illustration of alternative scenarios of 8*x*–2*x* hybrid polyploid speciation, in contrast with the ‘contemporary sympatric speciation’ hypothesis (H1). Under H1 with *F*. *vesca* ssp. *bracteata* of the Av subgenome being the 2*x* progenitor, four chromosomes of the decaploid are expected from the Av subgenome. Under the hypothesis of ‘ancient hybrid speciation’, four chromosomes are expected from the Bi (H2.A), B1 (H2.B) or B2 subgenome (H2.C), assuming that the decaploid originates from hybrid polyploid speciation between an ancestral 8*x* progenitor and a 2*x* progenitor of the allopatric *F*. *iinumae* of the Bi subgenome, or an extinct *F*. *iinumae*‐like 2*x* progenitor of the B1 or B2 subgenome, respectively. Under the ‘cryptic diversity’ hypothesis, the 2*x* progenitor possesses a previously unrecognized (U) subgenome, which could be closely related to the B subgenomes (solid line) or sister to both the Av and B subgenomes (dashed line).

Here we evaluated the evolutionary origin of a high‐order polyploid, *Fragaria cascadensis*, which is a recently discovered decaploid strawberry (Hummer, 2012), endemic to the Oregon Cascades (Fig. 1a). Allopatric populations exist at high elevations (1000–1750 m; Hummer, 2012; M. S. Dillenberger, pers. comm.), but at the lower margin of its distribution, *F*. *cascadensis* is sympatric with the western octoploid subspecies *F*.* virginiana* ssp. *platypetala* and diploid *F*. *vesca* ssp. *bracteata* (Fig. 1a; Staudt, 1999). However, pentaploid hybrids have never been reported in Oregon. All three species are sexually polymorphic, where populations contain female, male and/or hermaphrodite plants (Liston *et al*., 2014; T‐L. Ashman, pers. obs.). Sex expression, and in particular dominant male sterility, is inherited from the maternal parent in sympatric diploid and octoploid species, but is carried on different chromosomes (IV‐Av in Oregon *F*. *vesca* ssp. *bracteata*; Tennessen *et al*., 2013; VI‐B1 in *F*.* virginiana* ssp. *platypetala*; N. Wei, R. Govindarajulu, J. A. Tennessen, A. Liston, T‐L. Ashman, in review), with recessive male sterility also being observed in the diploid *F*. *vesca* ssp. *bracteata* (VI‐Av; Ashman *et al*., 2015). Thus, sex‐determining regions (SDRs) are among the most dynamic and distinguishing components of *Fragaria* genomes. The genetic basis of sex expression in the decaploid is not yet known, but a shared SDR with other mapped *Fragaria* species would provide additional evidence of ancestry independent of phylogeny.

The present‐day (partial) sympatry (Fig. 1a) has given rise to the hypothesis that *F*. *cascadensis* originated from hybrid polyploid speciation between the two sympatric congeners (Hummer, 2012). In this study, we contrasted this ‘contemporary sympatric speciation’ hypothesis between the octoploid and the diploid (H1; Fig. 1b) with competing hypotheses that recognize older and allopatric origins (H2–H3; Fig. 1b). In the light of potential contemporary allopatry between polyploids and progenitors and potential extinction of progenitors, we proposed hypotheses of ‘ancient hybrid speciation’, in which the putative diploid progenitor is currently allopatric (i.e. *F*. *iinumae*, distributed in Japan and Sakhalin Island, Russia; H2.A) or extinct (i.e. unknown *F*. *iinumae*‐like diploid with the B1 or B2 subgenome; H2.B and H2.C). Alternatively, the diploid progenitor contributing to the decaploid formation may possess a previously undetected subgenome; we refer to this possibility as the ‘cryptic diversity’ hypothesis (H3; Fig. 1b). To differentiate these hypotheses, we performed linkage mapping‐assisted subgenome inference of the decaploid in a phylogenetic context of previously characterized *Fragaria* genomes. We then statistically evaluated these evolutionary scenarios using topology‐based hypothesis testing. Lastly, to provide independent insights into the ancestry based on SDR, we incorporated the sex phenotype into the linkage map.

## Materials and Methods

### Experimental cross population

To obtain the linkage map of *Fragaria* *cascadensis* Hummer, an F_1_ mapping population was derived from two parental plants collected from the wild in Oregon (Linn County; 44.404°N, 122.076°W). As *F*. *cascadensis* is an outcrossing subdioecious species with high heterozygosity, an F_1_ cross is sufficient and effective to avoid inbreeding depression in linkage mapping. Seeds of the experimental cross (*N *=* *96) were sowed in a custom soil mixture (2 : 1, Fafard 4 : sand top‐dressed with Sunshine Redi‐earth Plug & Seedling; Sun Gro Horticulture, Agawam, MA, USA), and grown under a 14‐h photoperiod and 21 : 16°C, day : night temperatures in a growth chamber at the University of Pittsburgh for 11 wk. We harvested one leaf from each seedling (*N *=* *93) for DNA extraction before transplanting into 200‐ml pots in a mixture of 2 : 1 : 1, Fafard 4 : sand : perlite, and growing at 21 : 10°C in a glasshouse from February to May in 2016. A random subset (*N *=* *45) of the F_1_ progeny was selected for targeted sequence capture.

For these 45 F_1_ plants, we assessed sex expression as in previous work (Spigler *et al*., 2008). Male function was determined by pollen production, and a plant was scored as ‘male fertile’ if possessing plump, dehiscing anthers or ‘male sterile’ if possessing small, vestigial anthers. Female function was scored as the proportion of hand‐pollinated flowers producing fruit, but as a consequence of the asynchronous flowering, the data were collected only on 28 progeny.

### Targeted sequence capture

Our capture design of *Fragaria* baits v.2.0 (20 000 100‐bp probes; 1 × tiling density) aimed to anchor all homeologous chromosomes of the polyploid genome. Baits were designed from the reference *Fragaria* *vesca* L. genome assembly v.2.0 (Fvb, 208.9 Mb; Tennessen *et al*., 2014) that contains chromosomes I–VII and unanchored scaffolds of ≥ 10 kb (Fvb0), unassembled scaffolds (< 10 kb each) in *F*. *vesca* genome assembly v.1.0 (Shulaev *et al*., 2011), diploid *Fragaria* *iinumae* Makino contigs (genome size of 199.6 Mb; Hirakawa *et al*., 2014) that show no homology to the *F*. *vesca* genome, and octoploid linkage groups of *Fragaria* *chiloensis* (L.) Mill. and *Fragaria* *virginiana* Duchesne ssp. *virginiana* (Tennessen *et al*., 2014), as well as bacterial artificial chromosomes (BACs; J. A. Tennessen *et al*., unpublished) in the vicinity of the SDR in *F*. *virginiana* ssp. *virginiana* (Spigler *et al*., 2011). These custom‐designed baits (see details in Supporting Information Methods S1) were synthesized using MYbaits (MYcroarray, Ann Arbor, MI, USA).

DNA was isolated from silica‐dried leaves of the parents and 45 F_1_ progeny by Ag‐Biotech (Monterey, CA, USA), and sheared to *c*. 250 bp using a Covaris LE220R focused‐ultrasonicator at Profile Genomics (Alameda, CA, USA). We constructed individually indexed genomic libraries using the NEBNext Ultra DNA Library Prep Kit (New England BioLabs, Ipswich, MA, USA) according to the manufacturer's instructions (Methods S2). These 47 genomic libraries were then target enriched following the MYbaits protocol (Methods S2), and sequenced using a 1/3 lane of 2 × 150 bp on an Illumina HiSeq 3000 (San Diego, CA, USA) at the Center for Genome Research and Biocomputing at Oregon State University.

### Genotype calling and linkage mapping

De‐multiplexed, paired‐end reads were filtered against potential adaptor contamination and low‐quality bases using Trimmomatic v.0.35 (LEADING:20, TRAILING:20, SLIDINGWINDOW:5:20, MINLEN:50; Bolger *et al*., 2014), and merged using Pear v.0.9.6 (overlap of ≥ 20 bp; Zhang *et al*., 2014). We kept both successfully merged (single‐end) reads and unmerged paired‐end reads for subsequent analyses. These reads were first aligned to Fvb using Bwa v.0.7.12 (Li & Durbin, 2009). The unaligned reads were extracted from the resultant Bam files using samtools v.1.3 (Li *et al*., 2009), and converted back to Fastq files using Picard v.1.141 (http://broadinstitute.github.io/picard/) for a second round of alignment to the combined genomic data set of unassembled scaffolds of *F*. *vesca* genome v.1.0, *F*. *iinumae* contigs and *F*. *virginiana* ssp. *virginiana* BACs. The mpileup files from the two rounds of alignment were used for polyploid genotype calling with Polimaps (Tennessen *et al*., 2014), in which heterozygous and homozygous loci were identified with the default parameters, except for a per‐individual depth of ≥ 40× for the decaploid.

We created the linkage map using onemap (Margarido *et al*., 2007) in R v.3.3.0 (R Core Team, 2016). Maternal and paternal maps were examined separately using single nucleotide polymorphisms (SNPs) segregating in only one parent. Initial assignment to linkage groups (LGs) was based on a logarithm of odds (LOD) threshold of 5. The relative positions along LGs were then determined by adding SNPs using a touchdown LOD score from 5 to 4 and the Kosambi mapping function. Linkage groups of at least five SNPs were considered for subgenome inference. Homologous LGs were identified using SNPs segregating in both parents and their phylogenetic placement (see section ‘Phylogenetic inference of subgenome origins’).

To locate the region influencing male fertility, we treated male function as a dominant Mendelian locus (i.e. heterozygote coded as ‘ab’ for ‘male sterile’ and homozygote ‘aa’ for ‘male fertile’), and mapped the binary male function along with targeted capture genotypes to LGs as in Tennessen *et al*. (2013).

### Phylogenetic inference of subgenome origins

To infer subgenome origins, we treated each LG as a taxon in the phylogenetic context of *Fragaria* species, as each LG has the potential to have a unique phylogenetic placement. To do this with polimaps, we first searched for the quality‐filtered reads that contained LG SNPs (referred to as LG sequences) for *F*. *cascadensis* (‘Fcas’) and for the proposed octoploid progenitor *F*.* virginiana* ssp. *platypetala* (‘Fvp’; N. Wei, R. Govindarajulu, J. A. Tennessen, A. Liston, T‐L. Ashman, in review). Consensus sequences were made from the BAM files of LG sequences aligned to Fvb. Second, we obtained consensus LG sequences of previously published octoploids by quality filtering and aligning the single‐end reads of targeted capture to Fvb in *F*. *chiloensis* (‘Fchil’) and *F*. *virginiana* ssp. *virginiana* (‘Fvirg’) (Tennessen *et al*., 2014). To reduce taxon numbers in the phylogeny, we considered a pair of homologous maternal and paternal LGs as a single taxon for nonfocal polyploids (‘Fchil’ and ‘Fvirg’), as these homologous LGs have previously shown consistent subgenome origins (Tennessen *et al*., 2014). Third, for diploid *Fragaria* species, we used available whole‐genome shotgun sequences, including *F*. *vesca* ssp. *bracteata* (MRD30 and MRD60; Tennessen *et al*., 2013; LNF23; Tennessen *et al*., 2014), *F*. *iinumae*,* Fragaria mandshurica* Staudt and *Fragaria viridis* Duchesne (Tennessen *et al*., 2014), and the genome of *F*. *vesca* ssp. *vesca* (Shulaev *et al*., 2011). Consensus sequences were derived from VCF files by aligning each species to Fvb. Last, we performed multiple sequence alignments of polyploid LG sequences and diploid genomic sequences based on their alignment positions to Fvb. Phylogenetic inference used the maximum likelihood (ML) method with the GTR+Γ model in RAxML v.8.0.26 (Stamatakis, 2014). Confidence in node support was determined with 100 bootstrapping replicates. We built the ML tree for each of chromosomes I–VII rooted with *Rubus coreanus* Miq. (Hyun *et al*., 2014).

### Hypothesis testing of alternative evolutionary histories

To test the hypotheses concerning the evolutionary origin of *F*. *cascadensis* (Fig. 1b), we compared the ML trees of constraint topologies, each representing one hypothesis, with the corresponding ML unconstrained trees. Under the ‘contemporary sympatric speciation’ hypothesis (H1), the 10*x F*. *cascadensis* originates from the current sympatric 8*x F*. *virginiana* ssp. *platypetala* (of the Av, Bi, B1 and B2 subgenomes) and 2*x F*. *vesca* ssp. *bracteata* (of the Av subgenome). In this scenario, four of the 10 chromosomes, for each of chromosomes I–VII, of the decaploid are expected to derive from the Av subgenome, and two from each of the Bi, B1 and B2 subgenomes. Thus, H1 can be formulated as a constraint topology of (4,Av),((2,Bi),(2,B1),(2,B2)), in which numbers represent the count of LGs of *F*. *cascadensis*, and letters correspond to the subgenome clades. Specifically, the Av clade consisted of *F*. *vesca* ssp. *vesca* and *F*. *vesca* ssp. *bracteata* (MRD30, MRD60, LNF23), and the Bi, B1, B2 clades of the corresponding octoploid LGs of *F*. *chiloensis* and *F*. *virginiana* ssp. *virginiana* (Tennessen *et al*., 2014). Under the ‘ancient hybrid speciation’ hypothesis (H2), *F*. *cascadensis* originates from an 8*x* progenitor ancestral to extant octoploids, and a 2*x* progenitor of the allopatric *F*. *iinumae* with the Bi subgenome (H2.A), or an extinct *F*. *iinumae*‐like diploid that possessed the B1 (H2.B) or B2 subgenome (H2.C). Thus, H2.A–C can be formulated as (2,Av),((4,Bi),(2,B1),(2,B2)), (2,Av),((2,Bi),(4,B1),(2,B2)) and (2,Av),((2,Bi),(2,B1),(4,B2)), respectively. Under the ‘cryptic diversity’ hypothesis (H3), the 2*x* progenitor possessed a previously undetected subgenome, the constraint topology of which follows (2,Av),((2,Bi),(2,B1),(2,B2)),(2).

For each hypothesis, we built ~six constraint trees by alternating the combinations of homologous LGs in different subgenome clades, for each of chromosomes I–VII (Table S1). The best ML trees with constraint topologies were compared against the ML unconstrained trees using the Shimodaira–Hasegawa (SH) test in RAxML and the approximately unbiased (AU) test in Consel v.0.20 (Shimodaira & Hasegawa, 2001).

## Results

### Targeted sequence capture

Multiplexed targeted capture yielded on average 2.73 million paired‐end reads per individual (range 1.79–4.30 million) for *F*. *cascadensis* (data availability: National Center for Biotechnology Information, PRJNA382418). We aligned the capture reads of the parents and 45 F_1_ progeny to Fvb and the additional genomic data set consisting of unassembled scaffolds of *F*. *vesca* genome v.1.0, *F*. *iinumae* contigs and *F*. *virginiana* ssp. *virginiana* BACs. We found that on‐target capture reached 1735 kb, indicating that 86.7% of the targeted regions were successfully enriched (Figs S1 and S2). These on‐target sequences had a median depth of 32× per individual. In addition, we obtained off‐target sequences of 11 432 kb but with low coverage of 4× per individual. Overall, 1685 kb with a median depth of ≥ 40× per individual was retained for genotype calling and linkage mapping.

### Decaploid linkage map

We obtained 3326 maternal and 1253 paternal LG SNPs, as well as 628 biparental LG SNPs. Initial LG assignment resulted in 35 maternal and 41 paternal LGs. We focused on the LGs of at least five SNPs (35 maternal and 37 paternal) for map refinement. In principle, LGs harboring large gaps (≥ 35 cM) were subdivided into separate LGs, and redundant LGs were then rejoined according to their placement in a preliminary phylogenetic analysis and their alignment positions to Fvb.

**Figure 2:**
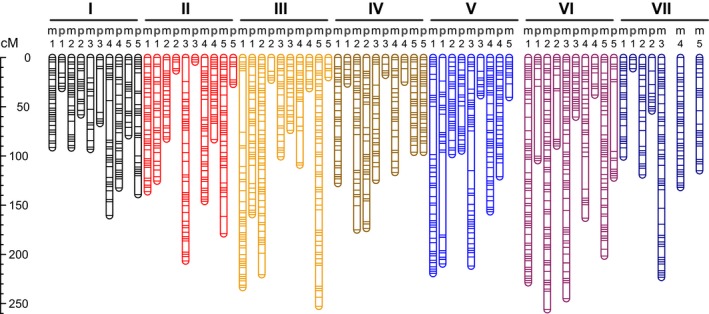
Linkage map of the decaploid *Fragaria cascadensis* derived from targeted sequence capture. Maternal (m) and paternal (p) linkage groups (LGs) are indicated at the top of the map. For each of chromosomes I–VII, five maternal and five paternal LGs are expected for the decaploid, as shown by the numbers (1–5). Four paternal LGs are missing from the final map, one in chromosome V (p‐5) and three in chromosome VII (p‐3, p‐4 and p‐5). Details of marker genetic positions are provided in Supporting Information Table S3.

The final maternal linkage map consisted of 35 LGs (*n *=* *5*x*,* x *=* *7; Fig. 2; Table S2), as expected for a decaploid. These maternal LGs varied in SNPs from 13 to 220, averaging 95 (median = 91; Table S2). Within each LG, most of the SNPs (90.8% on average) had a linkage map position consistent with their Fvb chromosome designation, whereas 5.8% of SNPs were from nonhomeologous chromosomes, probably indicative of translocations or assembly errors in Fvb (Table S2; Fig. S3). The proportion of SNPs derived from the unanchored scaffolds (Fvb0) and unassembled scaffolds (of < 10 kb each) in *F*. *vesca* genome v.1.0, and from *F*. *iinumae* contigs showing no homology to the *F*. *vesca* genome, was minimal for each LG, on average 0.2%, 2.0% and 1.1%, respectively (Table S2; Fig. S3). In addition, the Fvb physical positions of these LG SNPs (Table S3) revealed potential intrachromosomal inversions or assembly errors in Fvb, especially in chromosome II (e.g. Fcas‐II‐m‐4; Fig. S3). Overall, the maternal map (Fig. 2; Table S2) spanned a cumulative genetic length of 5358.8 cM, corresponding to a physical size of 966.8 Mb (considering only SNPs with Fvb chromosome designations), and individual LGs ranged from 40.5 cM (7.71 Mb) to 256.1 cM (38.02 Mb).

For the paternal map, the more homozygous genetic background of the male parent used in the experimental cross resulted in fewer and shorter LGs, as well as fewer SNPs per LG (Figs 2 and S3; Table S2). This probably reflected the difference in natural genetic variation between strictly outcrossing females and self‐compatible hermaphrodites. One paternal LG of chromosome V and three of chromosome VII were missing from the final linkage map (Fig. 2). Paternal LGs ranged from 4.5 cM (1.38 Mb) to 209.7 cM (28.33 Mb) (Table S2). The cumulative genetic length (2274.3 cM) was 2.4 times shorter than that maintained by the maternal map, with the physical size (567.4 Mb) being 1.7 times smaller. This magnitude of maternal–paternal difference in genetic length was comparable to that in LG SNPs. Paternal LGs possessed an average of 40 SNPs (range 5–99). However, similar to maternal LGs, most SNPs (90.7%) showed expected Fvb chromosome designations (Table S2; Fig. S3).

### Phylogenetic and subgenome inference of linkage groups

**Figure 3:**
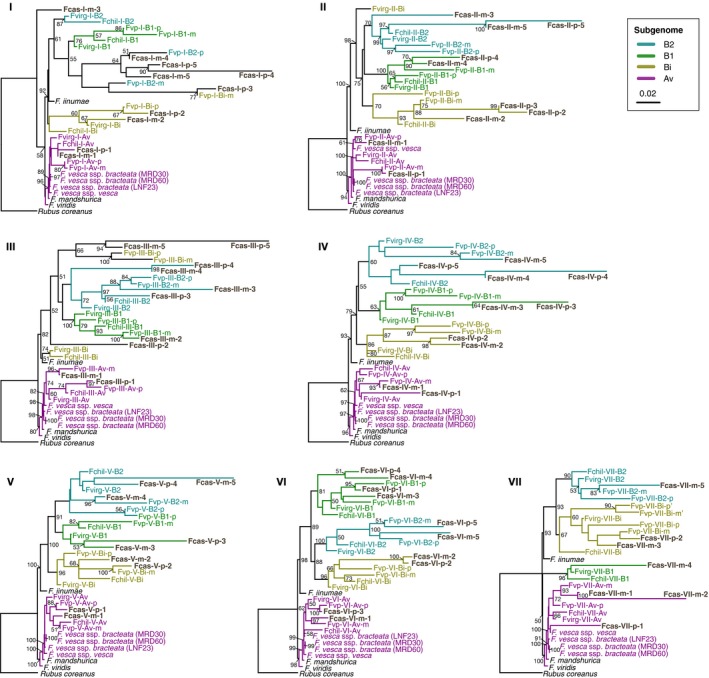
Maximum‐likelihood (ML) phylogenies of chromosomes I–VII for subgenome inference. The ML tree tips correspond to linkage groups (LGs) for polyploids and species for diploids. For the focal 10*x Fragaria cascadensis* (in bold), individual LGs are named after species (‘Fcas’), chromosome (I–VII), maternal or paternal map (m or p) and LG number (1–5). For the proposed 8*x* progenitor *Fragaria virginiana* ssp. *platypetala* (‘Fvp’), the LG designation follows species, chromosome, subgenome (Av, Bi, B1 and B2) and maternal or paternal map. In chromosome VII, however, the Bi and B1 subgenomes could not be unambiguously discriminated. For other previously studied 8*x Fragaria chiloensis* and *F*. *virginiana* ssp. *virginiana*, maternal and paternal homologous LGs were combined, and are represented following species (‘Fchil’ or ‘Fvirg’), chromosome and subgenome. The clades of individual subgenomes are color coded. Numbers associated with branches are ML bootstrap support values ≥ 50% from 100 replicates.

The nucleotide supermatrix was composed of 34 taxa (eight diploids, ten LGs for the decaploid ‘Fcas’, eight LGs for the octoploid ‘Fvp’, four homolog‐combined LGs for the octoploid ‘Fvirg’ and four for ‘Fchil’) and 166 580 characters with 3625 parsimony‐informative sites (PISs) on average for chromosomes I–VII, ranging from 118 884 (PIS = 2298; chromosome I) to 259 593 characters (PIS = 6611; chromosome VI). The ML unconstrained trees frequently discovered two out of the 10 LGs residing within the Av clade across chromosomes (except VII; Fig. 3). In addition, the Av LGs of the decaploid were mostly grouped with those of the octoploids, rather than with the proposed diploid progenitor *F*. *vesca* ssp. *bracteata*.

The relationships of the Bi, B1 and B2 clades varied among chromosomes I–VII (Fig. 3). First, the diploid *F*. *iinumae*, which was previously recovered as sister to the Bi LGs in octoploids (Tennessen *et al*., 2014), was found to be sister to the group consisting of all or most B clades (Bi, B1 and B2) here, based on broader genomic capture and the inclusion of both variable and invariable sites. Second, a polyphyletic placement of polyploid LGs occurred among the B clades in four chromosomes (I–III and VII). For instance, in chromosome II, one pair of homologous LGs of *F*. *cascadensis* (Fcas‐II‐m‐3 and Fcas‐II‐p‐3) was recovered at clades of disparate subgenome origins (B2 and Bi, respectively; BS = 75%; Fig. 3), indicative of potential genomic exchange between these two subgenomes. Likewise, the Bi LG of *F*. *virginiana* ssp. *virginiana* (Fvirg‐II‐Bi) was recovered as sister to all the remaining B clades (BS = 98%; Fig. 3). In chromosome III, the Bi LGs of the decaploid and the proposed octoploid progenitor (‘Fvp’) were not grouped with other LGs from the Bi clade, but instead sister to the B1 and B2 clades (Fig. 3). Third, the relationship between the B1 and B2 clades remained difficult to resolve unequivocally, a result of the similarity between the two subgenomes (Tennessen *et al*., 2014; Sargent *et al*., 2016). These two clades had low BS support in some cases (e.g. < 50% in chromosomes II and III; Fig. 3). Particularly in chromosome I, the B1 and B2 LGs of the decaploid were not separated, and were nested within the same clade with the B2 LGs of *F*. *virginiana* ssp. *platypetala*, together being successively sister to the B1 and B2 clades with weak to moderate support (BS = 55% and 61%, respectively; Fig. 3).

Some LGs of the B subgenomes were recovered as closely related to the Av clade in chromosomes I and VII (Fig. 3), albeit with low support (BS ≤ 50%). To assess these unexpected phylogenetic positions, we performed post hoc tests of introgression (Table S4) as in Tennessen *et al*. (2014), and found a considerable amount of introgression from the Av to B subgenomes and from the outgroup in these LGs. In chromosome I, one maternal LG (Fcas‐I‐m‐3) of *F*. *cascadensis* was placed as sister to other *Fragaria* (Fig. 3), in which 58% of its phylogenetic sites were similar to those of the outgroup species (*R*. *coreanus*,* F*. *viridis* and *F*. *mandshurica*), instead of the ingroup species, *F*. *vesca* (ssp. *bracteata* and ssp. *vesca*) and *F*. *iinumae* (Table S4). Introgression from the Av to B subgenomes was detected in chromosome VII, where LGs of the B subgenomes, Fcas‐VII‐m‐2 and Fcas‐VII‐m‐4, had on average 25% of the sites shared with *F*. *vesca*.

### Topology testing of alternative evolutionary histories

Both the AU and SH tests (Table 1) rejected the hypothesis of ‘contemporary sympatric speciation’ (H1; Fig. 1b) across all chromosomes (I–VII). These tests, however, supported the ‘ancient hybrid speciation’ hypothesis (H2) for five of the seven chromosomes, as the corresponding constraint topologies generated statistically similar likelihoods to the ML unconstrained trees (Table 1). The two chromosomes (I and VII) that rejected H2 did not support any of the alternative evolutionary hypotheses, because some B subgenome LGs resolved as more closely related to the Av clade than the B clades. Within H2, the AU and SH tests did not favor a particular evolutionary scenario among H2.A–C (Table 1), and this is probably caused by the varied relationships among the B clades. However, for the ML unconstrained trees (Fig. 3), more chromosomes agreed with the expectation of H2.B or H2.C instead of H2.A. The topology tests did not reject the ‘cryptic diversity’ hypothesis (H3) for chromosomes III and V (Table 1), despite having lower likelihoods compared with H2.

**Table 1:** Results of the approximately unbiased (AU) tests and Shimodaira–Hasegawa (SH) tests for alternative evolutionary hypotheses concerning the decaploid origin

	
	Chromosome	I	II	III	IV	V	VI	VII
ML unconstrained tree	−Log_e_ *L*	233 371	376 083	259 566	298 888	315 852	521 620	321 191
	(AU *P*)	(0.999)	(0.846)	(0.879)	(0.953)	(0.839)	(0.996)	(0.997)
‘Contemporary sympatric speciation’ hypothesis								
**H1** (4,Av),((2,Bi), (2,B1),(2,B2))	Delta −log_e_ *L*	185**	81**	49*	24*	20**	58**	51**
	(AU *P*)	(< 0.001)	(< 0.001)	(0.004)	(0.019)	(0.003)	(0.001)	(0.004)
‘Ancient hybrid speciation’ hypothesis								
**H2.A** (2,Av),((4,Bi), (2,B1),(2,B2))	Delta −log_e_ *L*	163**	**17**	**27**	20*	**17**	67**	208**
	(AU *P*)	(< 0.001)	**(0.160)**	**(0.150)**	(0.023)	**(0.153)**	(0.004)	(< 0.001)
**H2.B** (2,Av),((2,Bi), (4,B1),(2,B2))	Delta −log_e_ *L*	84**	71**	**15**	**14**	**6**	**0**	214**
	(AU *P*)	(< 0.001)	(< 0.001)	**(0.268)**	**(0.109)**	**(0.304)**	**(0.996)**	(< 0.001)
**H2.C** (2,Av),((2,Bi), (2,B1),(4,B2))	Delta −log_e_ *L*	100**	64**	**30**	**0**	**0**	43**	182**
	(AU *P*)	(0.001)	(< 0.001)	**(0.049)**	**(0.953)**	**(0.839)**	(0.003)	(< 0.001)
‘Cryptic diversity’ hypothesis								
**H3** (2,Av),((2,Bi), (2,B1),(2,B2)),(2)	Delta −log_e_ *L*	185**	87**	**27**	19*	**17**	15*	259**
	(AU *P*)	(< 0.001)	(< 0.001)	**(0.147)**	(0.063)	**(0.082)**	(0.017)	(< 0.001)

Constraint tree topologies were formulated in terms of the number of chromosomes (or linkage groups more precisely) of *Fragaria cascadensis* in the clade of the Av, Bi, B1, B2 or a previously undetected subgenome (‘cryptic diversity’ hypothesis), respectively. −Log_e_ *L*: negative log likelihood; Delta −log_e_ *L*: the difference in −log_e_ *L* between the best ML constraint tree of each hypothesis and the ML unconstrained tree for each of chromosomes I–VII. The *P* values of the AU tests are given within the parentheses, whereas the *P* values of the SH tests are denoted with asterisks: *, *P *<* *0.05; **, *P *<* *0.01. Hypotheses that cannot be rejected by the AU or SH tests are highlighted in bold. ML, maximum likelihood.

### Mapping sex expression

Male function segregated in a 1 : 1 ratio in 43 flowering F_1_ progeny out of the 45 in targeted sequence capture (male sterile : fertile = 20 : 23; χ^2^ = 0.093; *P *=* *0.760), and in the 28 F_1_ progeny assessed for female function (male sterile : fertile = 11 : 17; χ^2^ = 0.893; *P *=* *0.345). Male sterility was mapped to a ~* *1‐Mb region between 36.529 and 37.547 Mb on Fcas‐VI‐m‐4 of the B1 subgenome in chromosome VI. Of the 28 plants scored for female function, only three were female sterile (of < 5% fruit set, *sensu* Spigler *et al*., 2008), precluding mapping of female function as a qualitative trait.

## Discussion

The evolutionary origins of high‐order polyploids can reveal fundamental processes in speciation. Our phylogenetic analyses of subgenomes of a decaploid wild strawberry consistently rejected the seemingly parsimonious hypothesis of ‘contemporary sympatric speciation’ between octoploid and diploid congeners. Instead, most chromosomes supported a scenario of ‘ancient hybrid speciation’, particularly H2.B or H2.C (Fig. 1b), between an ancestral 8*x* progenitor and a presumably extinct, *F*. *iinumae*‐like 2*x* progenitor of the additional B1 or B2 subgenome (designated as Bc here for ‘cascadensis’), with the former being the maternal progenitor, as discussed later. Based on the present geographic distribution of the closest 2*x* relative of the B subgenomes (*F*. *iinumae*; Fig. 1a) and the closest 2*x* relative of the Av subgenome (*F*. *vesca* ssp. *bracteata*; northwestern North America), we speculate that the species contributing the B1, B2 and Bc subgenomes inhabited Beringia during the Pleistocene. This proposition is supported by the only fossil evidence of *Fragaria*, a 2.9‐Myr‐old achene from the Canadian Arctic at 76–77°N (Fig. 1a; Liston *et al*., 2014). We discuss the evidence for this ‘ancient hybrid speciation’ hypothesis from the perspectives of geography, genome and sex expression. We then discuss the use of this study as an exemplary starting step towards building much‐needed model systems of high‐order and/or old polyploids that have been, and remain to be, recognized.

### Present‐day sympatry unreliably predicts polyploid origin

Niche divergence and/or spatial segregation are thought to be necessary for limiting competition of polyploids with resident progenitor species that have high‐frequency advantage and are well adapted to the surrounding environments (Levin, 2003). Therefore, polyploid establishment can be accompanied by distribution shift, from initial sympatry with progenitor species to eventual parapatry or allopatry. Here, the 10*x F*. *cascadensis* is in partial sympatry with the 8*x F*. *virginiana* ssp. *platypetala* and the 2*x F*. *vesca* ssp. *bracteata*, but both the ML unconstrained trees and topology tests strongly rejected these two co‐occurring congeners being the putative progenitors. Spatial segregation between polyploids and progenitors has also been observed in the allotetraploid *Mimulus sookensis* (Benedict *et al*., 2012), which is mostly allopatric with the morphologically similar 2*x* progenitor *Mimulus* *nasutus*, while often in sympatry with the other 2*x* progenitor, *Mimulus* *guttatus*. In *Tragopogon*, the allotetraploid *Tragopogon* *castellanus* (Mavrodiev *et al*., 2015), which has an older origin than the newly formed allotetraploid *Tragopogon* *mirus* and *Tragopogon* *miscellus*, is allopatric with the paternal 2*x* progenitor, and spatially separated from the most closely related population of the maternal 2*x* progenitor despite broad sympatry in Spain. Together, these studies highlight the point that one cannot reliably infer the origins of polyploids that persist beyond the early stages of speciation from geography alone. However, recent studies comparing climatic niches of polyploids to one (e.g. Glennon *et al*., 2014) or two 2*x* progenitor species (e.g. Marchant *et al*., 2016), across a broad range of plant taxa, identified niche conservatism and intermediacy as the dominant patterns; that is, polyploids occupy the same or a subset of climatic niches relative to parental species. Although these findings seemingly contradict the theoretical expectations (Fowler & Levin, 1984, 2016; Oswald & Nuismer, 2011) regarding niche separation of polyploids from progenitors, niche similarity at broad geographic scales essentially underscores the importance of alternative mechanisms that operate at finer scales, such as spatial segregation, in the evolution of new polyploid lineages.

### Decaploid probably originated from historical hybrid speciation

Unlike other newly described polyploids that are of recent origin, including the 4*x T*. *mirus* and *T*. *miscellus* (Soltis *et al*., 2004), 6*x Senecio cambrensis* (Abbott & Lowe, 2004), 6*x Mimulus peregrinus* (Vallejo‐Marín *et al*., 2015) and 12*x Spartina anglica* (Ainouche *et al*., 2004), our results suggest an old origin for the 10*x F*. *cascadensis* that has remained cryptic until recently (Hummer, 2012). The ML analyses and topology tests provide evidence in support of the ‘ancient hybrid speciation’ hypothesis, albeit discriminating between H2.B and H2.C is constrained by the high similarity between the B1 and B2 subgenomes. The ‘ancient hybrid speciation’ may also underlie the formation of the other newly recognized decaploid in *Fragaria* (*Fragaria* *iturupensis*; Hummer *et al*., 2009), which is endemic to North East Asia (Iturup Island, Russia) and geographically close to the 2*x F*. *iinumae* (in Japan and Sakhalin Island, Russia). This species has also been reported as an octoploid (Staudt & Olbricht, 2008), and it is possible that both the 8*x* and 10*x* cytotypes occur. Based on two multiple‐labeled nuclear gene trees, Rousseau‐Gueutin *et al*. (2009) found that a 10*x* accession of *F*. *iturupensis* possessed homeologs from clades of the 2*x F*. *vesca* and 2*x F*. *iinumae*, respectively, with many more (> 4‐fold) from the latter clade supported by one gene tree. This raises the possibility that these two decaploid species separated by 6700 km across the North Pacific Ocean may have the same subgenomes and evolutionary origin.

The genomic composition and topology tests here, combined with the chloroplast phylogeny of *Fragaria* (Njuguna *et al*., 2013), enable us to envision some possible evolutionary scenarios for the focal decaploid and closely related octoploids. One plausible scenario involves hybrid polyploid speciation between a maternal, ancestral 8*x* progenitor of the Av, Bi, B1 and B2 subgenomes and a paternal, *F*. *iinumae*‐like 2*x* progenitor of the Bc subgenome (H2.B or H2.C). The phylogeny of the maternally inherited chloroplast (Njuguna *et al*., 2013) supports extant 8*x* and 10*x Fragaria* sharing an *F*. *vesca*‐like plastid genome, and this is also observed in *F*. *cascadensis* (M. S. Dillenberger *et al*., unpublished), thereby excluding *F*. *iinumae*‐like 2*x* species as the maternal parent. The formation of the decaploid probably occurred shortly after that of the ancestral octoploid, because of the close phylogenetic relationship between *F*. *cascadensis* and extant octoploids, which could not be fully resolved using the genome‐wide data here. Following origination probably in Beringia during the Pleistocene (Liston *et al*., 2014), the ancestral populations of the 8*x* and 10*x Fragaria* migrated to North and South America and diverged to occupy disparate environments (Staudt, 1999), with some staying in North East Asia (i.e. the 10*x* and potentially also 8*x F*. *iturupensis*).

Although the evolutionary hypotheses were formulated in the framework of 8*x*–2*x* hybrid polyploid speciation for testing the leading hypothesis (H1; Fig 1b), our data were unable to exclude other possible, more complex scenarios that could give rise to the same prediction as the ‘ancient hybrid speciation’ hypothesis (H2.B or H2.C). For instance, the decaploid might have originated from an *F*. *vesca*‐like 2*x* maternal progenitor of the Av subgenome hybridizing with an unknown, extinct octoploid that differed from all extant octoploids by possessing only the B subgenomes (Bi, B1, B2 and Bc). However, in this scenario the Av subgenome of the decaploid should be most closely related to *F*. *vesca*, but the ML unconstrained trees indicated otherwise, with the *F*. *cascadensis* Av subgenome being more closely related to extant octoploids (Fig. 3).

Other, albeit less parsimonious, possibilities that merit particular consideration include 4*x*–6*x* hybrid polyploid speciation. Tennessen *et al*. (2014) proposed a 4*x*–4*x* scenario for the 8*x Fragaria*, in which the maternal 4*x* progenitor probably arose from homoploid hybridization between two diploids of the Av and Bi subgenomes, respectively, and the paternal 4*x* progenitor from a diploid of the B1 subgenome hybridizing with a likely conspecific diploid of the B2 subgenome. There are two points in favor of their hypothesis: first, the B1 and B2 subgenomes are fairly similar; second, homoploid hybrids theoretically experience reduced reproductive isolation relative to heteroploid hybrids, by conforming to the 2 : 1 ratio of maternal to paternal genomic dosage required for normal endosperm development in seeds (Scott *et al*., 1998; Stoute *et al*., 2012). Likewise, under a 4*x*–6*x* scenario, the decaploid could have originated from the same, extinct maternal 4*x* progenitor of the extant octoploids, and an extinct, paternal 6*x* progenitor of the B1, B2 and Bc subgenomes, which was possibly a cytotype of the same paternal 4*x* progenitor. Compared with the 8*x*–2*x* scenario, although deviation from the 2 : 1 parental genomic ratio is less severe in the 4*x*–6*x*, the maternal genomic excess of the 8*x*–2*x* can benefit descendent fitness (i.e. seed viability; Stoute *et al*., 2012). Additional efforts to discriminate between these two scenarios of hybrid polyploid speciation are required.

### SDR provides some insights into decaploid origin

Like the 8*x Fragaria* studied to date, male sterility in the 10*x F*. *cascadensis* is dominant and maps to the same homeologous group (chromosome VI); however, it differs in subgenome and chromosomal position. Specifically, for the proposed 8*x* progenitor *F*. *virginiana* ssp. *platypetala*, male function maps to the same B1 subgenome as in *F*. *cascadensis* but at a chromosomal position closer to the middle (13 Mb; N. Wei, R. Govindarajulu, J. A. Tennessen, A. Liston, T‐L. Ashman, in review). By contrast, in the 8*x F*. *chiloensis*, male function is located at a similar distal end of a chromosome (37 Mb) but on a different (Av) subgenome (Tennessen *et al*., 2016), a location also observed in the natural hybrid, 8*x F*. ×*ananassa* ssp. *cuneifolia*, with *F*. *chiloensis* as the maternal parent (Govindarajulu *et al*., 2013). In *F*. *virginiana* ssp. *virginiana*, the location differs in both subgenome (B2) and chromosomal position (the start of a chromosome; Spigler *et al*., 2010). Also notable is that dominant male sterility in the partially sympatric 2*x F*. *vesca* ssp. *bracteata* maps to a different homeologous group, that is, chromosome IV (Tennessen *et al*., 2013). Although recessive male sterility of the diploid maps to chromosome VI, it is located in a different (Av) subgenome (Ashman *et al*., 2015). The observed variability could indicate SDR turnovers among these polyploids, but the absence of a shared location with other species is consistent with an older origin of the decaploid rather than a recent hybrid origin *in situ*, as revealed by the topology tests.

### Towards a unified understanding of high‐order and old polyploids

Our study enables a statistical evaluation of evolutionary hypotheses concerning the decaploid origin, and also provides insights into chromosomal structure as well as mapping of relevant phenotypes in a more unified manner, relative to other means such as GISH and FISH or phylogenetic networks. Cytological examinations with GISH and FISH have been used for inferring origins and chromosomal changes in both low‐ and high‐order polyploids of recent or old origin (e.g. Lim *et al*., 2000; Chester *et al*., 2012; Liu *et al*., 2016), but not for genome‐wide polyploid phylogeny or hypothesis testing. In addition, reticulate evolutionary histories of polyploids have been evaluated using nonbifurcating networks in some polyploid‐rich genera of low ploidies such as *Nicotiana* (Kelly *et al*., 2013), and of high ploidies such as *Cerastium* (≤ 12*x*; Brysting *et al*., 2007), *Primula* sect. *Aleuritia* (≤ 14*x*; Guggisberg *et al*., 2009) and *Viola* (≤ 18*x*; Marcussen *et al*., 2012). These studies using phylogenetic networks provided qualitative, post hoc interpretations of the origins of high‐order and/or old polyploids. By contrast, linkage group‐based phylogenetic trees used here permit an explicit statistical link between evolutionary hypothesis and test, which could be evaluated with mature phylogenetic methods of topology testing. Such approaches are still in their infancy for phylogenetic networks. We should point out, however, that whole LG concatenation may be phylogenetically misleading if substantial chromosomal rearrangements and/or gene flow occurs before or after polyploidy. In this study, the extent of chromosomal rearrangements was minimal for most chromosomes (Fig. S3). Our study did not allow robust estimation of gene trees (data not shown), as individual loci provided few phylogenetically informative sites. Despite these potential caveats, most sites of each LG should reflect correct phylogenetic history, and the subgenome origins of a polyploid can be independently and repeatedly reflected by individual homeologous groups (e.g. chromosomes I–VII here).

Compared with old polyploids, recently formed neopolyploids, especially the low‐order ones, are overrepresented as model systems for our understanding of polyploidy in plants (reviewed in Soltis *et al*., 2016). However, neopolyploids are evolutionarily labile and may have higher extinction rates (Soltis *et al*., 2014), and could be profoundly different from old polyploids with respect to the complexity of evolutionary histories, genomic dynamics and ecological consequences. Our study of the newly discovered, yet old decaploid can serve as an exemplary starting step towards building model systems of persistent polyploid lineages, through a unified framework of understanding evolutionary origin, genomic composition, chromosomal variation and linkage mapping. With this foundation, we may begin to address other fundamental questions such as recurrent formation, genomic plasticity and ecological adaptation.

## Author contributions

T‐L.A. and A.L. designed the research; N.W. performed the research; N.W. and J.A.T. carried out data analyses; N.W., J.A.T., A.L. and T‐L.A. wrote the manuscript.

## Supporting information

Please note: Wiley Blackwell are not responsible for the content or functionality of any Supporting Information supplied by the authors. Any queries (other than missing material) should be directed to the *New Phytologist* Central Office.

**Fig. S1** Mean per‐individual depth of coverage using baits designed from *Fragaria* chromosomes I–VII.
**Fig. S2** Mean per‐individual depth of coverage using baits designed from additional genomic data sets.
**Fig. S3** Plots of genetic position versus physical position of linkage group SNPs.
**Methods S1** Methodological details on capture baits design.
**Methods S2** Targeted sequence capture library preparation.

**Table S1** Topologies of constraint trees for hypothesis testing

**Table S2** Summary statistics of maternal and paternal linkage groups

**Table S3** Descriptions of all the markers on linkage groups

**Table S4** Post hoc tests of introgression in linkage group‐specific sequences
