# Supplementary material for: Present‐day sympatry belies the evolutionary origin of a high‐order polyploid
Source: New Phytol. 2017 Aug 3;216(1):279–90. doi: 10.1111/nph.14711 (PMC5637924; doi:10.1111/nph.14711)
Supplement: Supplementary file 1 — Fig. S1 Mean per‐individual depth of coverage using baits designed from Fragaria chromosomes I–VII. Fig. S2 Mean per‐individual depth of coverage using baits designed from additional genomic data sets. Fig. S3 Plots of genetic position versus physical position of linkage group SNPs. Methods S1 Methodological details on capture baits design. Methods S2 Targeted sequence capture library preparation. [file NPH-216-279-s001.pdf]

***New Phytologist* Supporting Information**

Article title: **Present-day sympatry belies the evolutionary origin of a high-order polyploid**

Authors: Na Wei, Jacob A. Tennessen, Aaron Liston and Tia-Lynn Ashman

Article acceptance date: 20 June 2017

The following Supporting Information is available for this article:

Fig. S1 Mean per-individual depth of coverage using baits designed from *Fragaria* chromosome I–VII.

Fig. S2 Mean per-individual depth of coverage using baits designed from additional genomic data sets.

Fig. S3 Plots of genetic position vs. physical position of linkage group SNPs.

Table S1 Topologies of constraint trees for hypothesis testing (see separate Excel file).

Table S2 Summary statistics of maternal and paternal linkage groups (see separate Excel file).

Table S3 Descriptions of all the markers on linkage groups (see separate Excel file).

Table S4 Post-hoc tests of introgression in linkage group-specific sequences (see separate Excel file).

Methods S1 Methodological details on capture baits design.

Methods S2 Targeted sequence capture library preparation.

Fig. S1 Mean per-individual depth of coverage using baits designed from *Fragaria* chromosome I–VII. This experimental cross of *Fragaria cascadensis* (*N* = 47) was sequenced using a 1/3 lane on an Illumina HiSeq 3000 (paired end 150 bp). The length of individual chromosomes (I–VII) was indicated according to *F*. *vesca* genome assembly v2.0. On average, 2350 baits were designed for individual chromosomes, ranging from 1745 (chromosome I) to 3242 (chromosome VI).

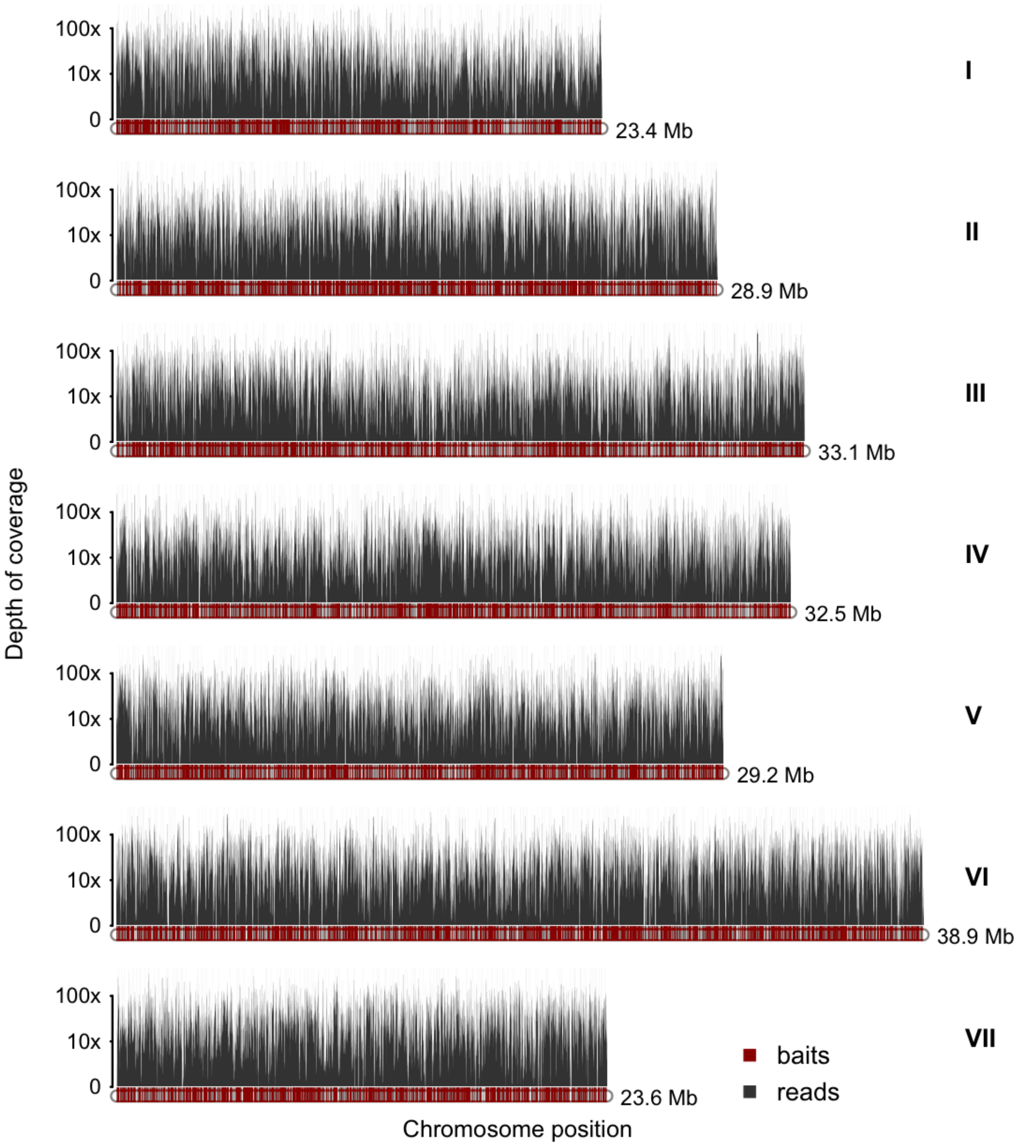

Fig. S2 Mean per-individual depth of coverage using baits designed from additional genomic data sets. These genomic data sets include un-anchored scaffolds over 10 kb (Fvb0) in *Fragaria vesca* genome assembly v2.0, un-assembled scaffolds (<10 kb each) in *F. vesca* genome assembly v1.0, *F*. *iinumae* contigs that show no homology to *F. vesca* genome, and octoploid *F*. *virginiana* ssp. *virginiana* BACs (bacterial artificial chromosomes) near the vicinity of its sex-determining region. The sequences of each data set (except Fvb0) were concatenated, and the corresponding cumulative length is indicated.

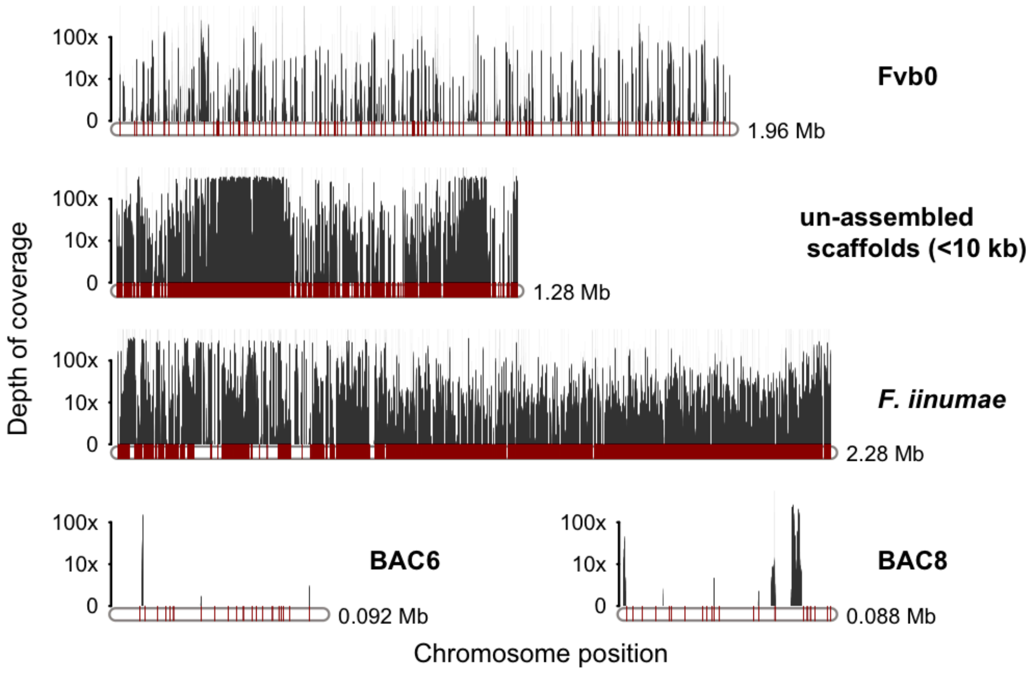

Fig. S3 Plots of genetic position vs. physical position of linkage group SNPs. Linkage groups (LGs) are organized according to chromosomes (I–VII, by column). For each of chromosome I–VII, five maternal and paternal LGs (1–5, by row) are expected. Maternal and paternal LGs are represented by red and grey color respectively. Most LG SNPs corresponded to Fvb chromosome designations, shown as solid dots. The LG SNPs that did not show expected Fvb chromosome designations are indicated as follows: those from non-homeologous chromosomes as open circles around the solid dots; those from un-anchored scaffolds (Fvb0) as reverse triangles (▽); those from un-assembled scaffolds of <10 kb each as cross signs (×); those from *F. iinumae* contigs as plus signs (+). The physical positions of these SNPs that did not show expected Fvb chromosome designations were extrapolated according to the relation (i.e. the slope) of cM vs. Mb of neighbouring markers, and their genetic positions were adjusted by increasing 10 cM to improve data visualization. Details of the genetic and physical positions of all the LG SNPs are provided in Table S3.

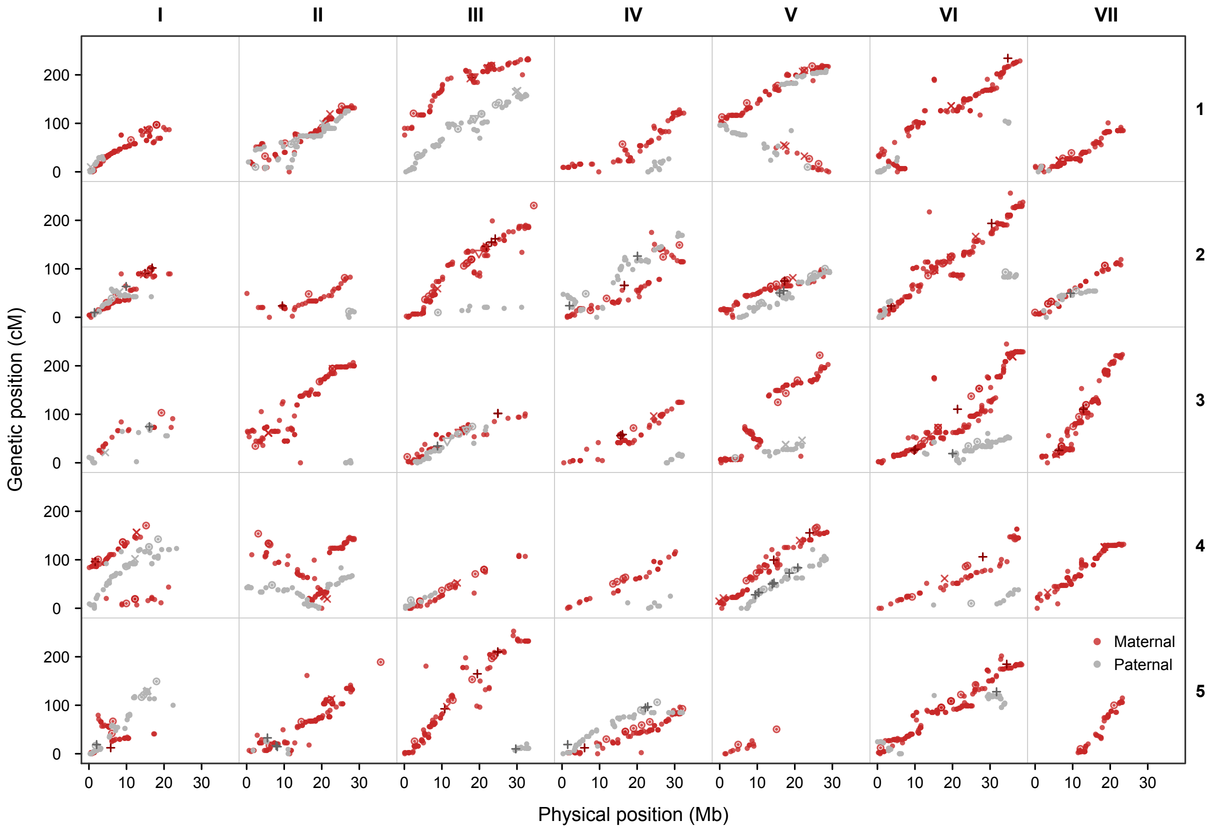

Methods S1 Methodological details on capture baits design

To achieve a good representation of the polyploid genome, we designed 20,000 100-bp capture probes (*Fragaria* baits v2.0) intended to anchor all homeologous chromosomes. Specifically, we first retained 7611 baits that were designed previously for *Fragaria* baits v1.0 (Tennessen *et al.*, 2013) from the reference *Fragaria vesca* genome assembly v1.1 (Genome Database for Rosaceae; https://www.rosaceae.org/) and were also found to harbor polymorphic loci in octoploid linkage maps of *F*. *chiloensis* and *F*. *virginiana* ssp. *virginiana* (Tennessen *et al.*, 2014). In contrast to the previous 1.5× tiling–that is, two baits flanking and one bait overlaying a central targeted polymorphism–in Tennessen *et al.* (2013), we adopted single bait design for selecting these 7611 baits and developing new bait sequences.

New baits were first designed from previously untargeted regions in the reference *F*. *vesca* genome assembly v2.0 (Fvb; Tennessen *et al.*, 2014) that includes seven chromosomes (I–VII) and un-anchored scaffolds over 10 kb (Fvb0). To do this, the reference genome assembly was fractured into contigs that were separated by a continuous stretch of ≥20 unknown bases. We searched for 100 bp candidate sequences in these contigs that were not targeted before, following the criteria of no unknown bases, no homopolymer of ≥6 bp, intermediate GC content of 30–70%, and unique sequences in the genome (BLAST e-value of $\leq 10^{-15}$ and percent identity cutoff of 85%). A single bait was retained for each contig, even if additional suitable candidates were detected. As a result, 8372 such baits were designed. Because these contigs were variable in length, the reference genome assembly was unlikely represented evenly. To overcome this, we then designed baits for all the remaining regions of ≥25 kb with no bait present, by searching for a candidate bait sequence every 25 kb; this added 583 more baits. In addition, 742 un-assembled scaffolds of *F*. *vesca* genome v1.0 (Shulaev *et al.*, 2011), which were shorter than 10 kb each and thus have not been incorporated into previous genome assemblies, were used to supplement our new set of *Fragaria* baits. In this respect, additional 1604 baits were designed with relaxed criteria of GC content (10–90%) and homopolymers (≤9 bp).

Furthermore, 1789 baits were developed from *F*. *iinumae* contigs (Hirakawa *et al.*, 2014; https://www.rosaceae.org/species/fragaria/fragaria_vesca/genome_v1.0) that did not show homology to *F*. *vesca* genome. Lastly, we obtained 41 baits from octoploid *F*. *virginiana* ssp.

virginiana bacterial artificial chromosomes (BACs; J. A. Tennessen *et al*., unpublished) that were in the vicinity of the sex-determining region (Spigler *et al.*, 2011).

Methods S2 Targeted sequence capture library preparation

We constructed DNA libraries using NEBNext Ultra DNA Library Prep Kit (New England BioLabs, Ipswich, MA, USA). Briefly, sheared dsDNA (100 ng) was first end repaired and ligated with universal adaptors. Adaptor-ligated DNA was size selected for averagely 350 bp using AMPure XP Beads (Beckman Coulter, Brea, CA, USA). Dual indexes (NEBNext Multiplex Oligos for Illumina) were then incorporated to each sample during PCR enrichment (of 6 cycles). After purification to remove primer dimers with AMPure XP Beads, the final individually indexed libraries were quantified using a Qubit 2.0 Fluorometer (Invitrogen, Carlsbad, CA, USA) and 2100 Bioanalyzer (Agilent, Santa Clara, CA, USA).

For targeted sequence capture, we pooled 23 or 24 genomic libraries at equal molar concentration in a single reaction for bait hybridization, with a total of two reactions for the experimental cross. Following MYbaits user manual v3.0 (http://www.mycroarray.com/pdf/MYbaits-manual-v3.pdf), 500-ng pooled libraries in a single reaction were incubated with the biotinylated baits at 65°C for 24 h. Hybridized bait–target products were recovered using Dynabeads MyOne Streptavidin C1 magnetic beads (Thermo Fisher Scientific, Waltham, MA, USA). Targeted sequences were then enriched using PCR of 15 cycles and purified with a 0.9× ratio of AMPure XP Beads. The final target-enriched individual pool was quantified using Qubit 2.0 and 2100 Bioanalyzer. We combined the two pools (47 total samples) at equal molarity for paired-end 150 bp sequencing using a 1/3 lane of an Illumina HiSeq 3000 at the Center for Genome Research and Biocomputing (CGRB) at Oregon State University.

References

Hirakawa H, Shirasawa K, Kosugi S, Tashiro K, Nakayama S, Yamada M, Kohara M, Watanabe A, Kishida Y, Fujishiro T *et al.* 2014. Dissection of the octoploid strawberry genome by deep sequencing of the genomes of *Fragaria* species. *DNA Research* **21**: 169-181.

Shulaev V, Sargent DJ, Crowhurst RN, Mockler TC, Folkerts O, Delcher AL, Jaiswal P, Mockaitis K, Liston A, Mane SP *et al.* 2011. The genome of woodland strawberry (*Fragaria vesca*). *Nature Genetics* **43**: 109-116.

Spigler RB, Lewers KS, Ashman T-L. 2011. Genetic architecture of sexual dimorphism in a subdioecious plant with a proto-sex chromosome. *Evolution* **65**: 1114-1126.

Tennessen JA, Govindarajulu R, Ashman T-L, Liston A. 2014. Evolutionary origins and dynamics of octoploid strawberry subgenomes revealed by dense targeted capture linkage maps. *Genome Biology and Evolution* **6**: 3295-3313.

Tennessen JA, Govindarajulu R, Liston A, Ashman T-L. 2013. Targeted sequence capture provides insight into genome structure and genetics of male sterility in a gynodioecious diploid strawberry, *Fragaria vesca* ssp. *bracteata* (Rosaceae). *G3: Genes, Genomes, Genetics* **3**: 1341-1351.
